# The Two Routes of Collective Psychological Ownership: Rights and Responsibilities Explain Intentions to Exclude Outsiders and Engage in Stewardship Behavior

**DOI:** 10.1177/01461672221129757

**Published:** 2022-10-26

**Authors:** Tom Nijs, Borja Martinovic, Maykel Verkuyten

**Affiliations:** 1 Utrecht University/Ercomer, The Netherlands

**Keywords:** collective psychological ownership, determination right, group responsibility, exclusion of outsiders, stewardship

## Abstract

People can have a sense of collective ownership of a particular territory, such as “our” country, “our” neighborhood, and “our” park. Collective psychological ownership is argued to go together with rights and responsibilities that have different behavioral implications. We found that collective psychological ownership leads to perceived determination right, and indirectly to the exclusion of outsiders from “our” place. Simultaneously, collective psychological ownership leads to perceived group responsibility, and indirectly to engagement in stewardship behavior. These results were found among Dutch adults, cross-sectionally in relation to their country (Study 1; *N* = 617) and a neighborhood (Study 2; *N* = 784), and experimentally in relation to an imaginary local park (Study 3; *N* = 384, Study 4; *N* = 502, both pre-registered). Our research shows that the feeling that a place is “ours” can, via perceived rights and responsibilities, result in both exclusionary and prosocial behavioral tendencies.

A sense of ownership is an important facet of people’s lives. It involves a psychological connection to what is owned, structures social situations, and defines social relationships in terms of who does, and does not have, the right to use, change, give away, or sell the things that are owned (Blumenthal, 2010). People can feel that something belongs to them personally (“mine”), and also that particular things belong to their ingroup (“ours”). This latter feeling is labeled collective psychological ownership (Pierce & Jussila, 2011; Verkuyten & Martinovic, 2017) and can be experienced in relation to territories, such as “our” country (Brylka et al., 2015; Selvanathan et al., 2020; Storz et al., 2020), “our” neighborhood (Toruńczyk-Ruiz & Martinovic, 2020), and “our” park (Peck et al., 2020; Preston & Gelman, 2020).

The importance of a sense of collective ownership has received very little attention in intergroup research. There is a large social psychological literature on social categorization with the related “us-them” thinking, but hardly any systematic theorizing and research on the nature and implications of thinking in terms of “ours” (Verkuyten & Martinovic, 2017). This is unfortunate because—as we will try to show—feelings of collective territorial ownership can play an important role in group dynamics. On a dark side, a sense of collective ownership can be a major source of exclusionary behavior, intergroup tensions, and territorial disputes and conflicts in the world (Toft, 2014). On a bright side, it can be involved in intragroup processes of cooperation, solidarity, and stewardship behavior (Hernandez, 2012). In the current article, we argue that collective psychological ownership of territory involves both perceived group rights and group responsibilities, and that these different aspects can have exclusionary and prosocial implications, respectively.

First, we posit that collective psychological ownership implies a perceived exclusive right to determine what happens with what is ours and who can use it (Merrill, 1998; Waldron, 1988). This determination right can serve as a basis for excluding non-owners, such as international migrants or those not living in “our” neighborhood. Second, we argue that collective psychological ownership is accompanied by perceived responsibility for taking care of what is “ours.” This perceived responsibility can increase stewardship behavior, for example, donating money or doing voluntary work for the benefit of maintaining or improving a particular territory (Hernandez, 2012).

In four studies among Dutch adults, we tested these propositions both cross-sectionally and experimentally in relation to three types of territories. Using cross-sectional data, we focused on collective psychological ownership in relation to the country in Study 1 and in relation to the neighborhood in Study 2. In experimental Studies 3 and 4, we tested our hypotheses in relation to a local park. All three territories are targets of collective psychological ownership (“ours”) because it is highly unlikely that people have the feeling that they personally own the country, neighborhood, or local park (“mine”). Yet, people might respond differently to things that directly impact them (neighborhood) compared to those with a broader (national) societal impact (Trope & Liberman, 2010), which allows us to examine the robustness of the findings. Thus, by testing the same model in relation to these different territorial targets of ownership and using different methods, manipulations, and measures, we aim to provide a conceptual replication that enhances our confidence in the theoretical propositions (Crandall & Sherman, 2016). Furthermore, whereas a few social psychologists have focused on the detrimental consequences of collective psychological ownership for intergroup relations (e.g., Nijs, Martinovic, et al., 2021; Selvanathan et al., 2020), organizational psychologists have mainly focused on involvement and investment in the target of ownership (Henssen et al., 2014; Pierce & Jussila, 2011). By examining both the rights and responsibilities of collective psychological ownership, we aim to offer a comprehensive picture and systematic investigation of the diverse implications for group dynamics. In considering the power of “ours” and by focusing on collective psychological ownership of territories, we try to make a novel contribution to social psychology and our understanding of the critical, but largely neglected, role that shared territories play in people’s thinking, feeling, and doing (Meagher, 2020).

## Collective Psychological Ownership

Societies function around a common understanding of ownership, as ownership organizes the physical environment and defines expectations, rights, and responsibilities that shape social interactions and relationships (Verkuyten & Martinovic, 2017). Even without being the legal owner, individuals can experience that something is owned by their group (Pierce & Jussila, 2011). This collective psychological ownership (“this is *ours*”) is based on a sense of “us” as proposed in self-categorization theory (Turner et al., 1987). According to this theory, people can understand themselves as a unique individual (personal self) and as a member of a group (group self), and these self-understandings are qualitatively different. A psychological change from the personal self to a group self implies a transformation of self-related terms and concerns: from personal self-esteem to collective self-esteem, personal efficacy to collective efficacy, personal responsibility to collective responsibility, personal interests to collective interest, and from personal ownership to collective ownership. Self-categorization theory argues that both the content and dynamics of these issues will be different as a function of whether they relate to the personal self or to a group self. And social psychological research has demonstrated that intergroup relations depend on the group self being salient and relevant; there is much empirical support for this, including neurological evidence (see Cikara & Van Bavel, 2014; Xiao et al., 2016). We focus on shared territories as the collective targets of ownership which can involve a sense of “ours” that is based on a sense of “us.” Furthermore, defining oneself in group terms is “essentially the precondition for all other dimensions of collective identity” (Ashmore et al., 2004, p. 84). Whereas collective psychological ownership implies self-categorization at the group level and the related sense of “us,” it is not dependent on a sense of ingroup attachment and commitment.

The role of territories has received little attention among social psychologists, while many social behaviors are territorially embedded (Meagher, 2020) and territories are central in many intergroup conflicts (Toft, 2014). As ownership of territory involves the use of a specific place with respect to others, territories are inherently social. Collective ownership claims of the country are frequently made in the political arena. Specifically right-wing populists argue that “this country is ours” or “we should take back our country” (Nijs, Martinovic, et al., 2021). Feelings of collective ownership are also relevant in a local context. Given the sometimes relatively high residential mobility between neighborhoods (Van Ham & Clark, 2009), questions of “to whom does this neighborhood belong?” are important. Also, collective ownership claims of local parks or community gardens influence social behaviors (Spierings et al., 2018).

## Collective Psychological Ownership and Exclusive Determination Right

Philosophers and legal scholars agree that ownership is accompanied by specific rights (Katz, 2008; Merrill, 1998; Snare, 1972; Waldron, 1988). Some argue that ownership comes with a bundle of rights, including the right to use one’s property, transfer it to others, and exclude others from using it (Snare, 1972). Others argue that the right to exclusion (Merrill, 1998), or exclusivity (Katz, 2008), is the central defining feature of ownership: owners have the right “to determine how the object shall be used and by whom” (Waldron, 1988, p. 39).

Empirical research has supported the link between ownership and perceived exclusive determination right, both at the personal and the collective level. Regarding personal ownership, pre-school children were found to understand that when someone controls the use of a toy, that person is probably the owner of it (Neary et al., 2009), and 6- to 8-year olds also apply this logic to ownership of ideas (Shaw et al., 2012). Regarding collective ownership, Dutch and British natives who believe the country is “theirs” were found to generally think that their ingroup has the exclusive right to determine matters that concern their country, for instance, who is allowed to enter (Nijs, Martinovic, et al., 2021).

The perception that “we” have an exclusive determination right can lead to the behavioral tendency to exclude outsiders. Collective psychological ownership implies group boundaries between owners and non-owners based on arguments, such as “we were here first” (autochthony) and “we used it and made it as it is today” (investment) (Verkuyten & Martinovic, 2017). Established inhabitants might perceive themselves to be the rightful owners of a territory and therefore to be entitled to exclude outsiders, such as international migrants or those not living in “our” neighborhood. Excluding outsiders from what is “ours” can be considered a self-evident consequence of the exclusive determination right, that is not considered unjust or discriminatory (Nijs, Martinovic, et al., 2021; Verkuyten & Martinovic, 2017). Research found that collective psychological ownership of the country and of the neighborhood is related to more negative attitudes toward outsiders (Brylka et al., 2015; Nijs, Martinovic, et al., 2021; Toruńczyk-Ruiz & Martinovic, 2020). In the current study, we examine the behavioral intention to exclude outsiders. Exclusionary behavior can be regarded an anticipatory defense response to prevent infringement of a group’s ownership (Brown et al., 2005). Taken together, we hypothesize that:

**Hypothesis 1 (H1):** Collective psychological ownership of a territory increases the behavioral tendency to exclude outsiders indirectly via higher perceived exclusive determination right.

## Collective Psychological Ownership and Group Responsibility

Next to an exclusive determination right, collective psychological ownership is typically accompanied by perceived responsibility (Furby, 1978; Peck & Shu, 2018; Wright, 2018). People do not only feel personally responsible for their individual ownership but can also feel that their group is responsible for what they collectively own. A general idea that “we should take care of what is ours” might make group members feel a moral obligation or duty to take care of what is theirs and might imply perceived normative pressure from fellow co-owners to take responsibility. Moreover, people can regard collective ownership as defining who they are as a group, which makes taking care of what they collectively own a way to maintain, protect, or enhance the group self: taking care of what is “ours” can be perceived as taking care of “ourselves” (Pierce & Jussila, 2011; Verkuyten & Martinovic, 2017). Although studies have shown that collective psychological ownership of products and jobs relates to personal responsibility (e.g., Kamleitner & Rabinovich, 2010), to our knowledge, no study has examined the link between collective psychological ownership and perceived group responsibility.

A sense of group responsibility can in turn manifest itself in the behavioral intention to engage in stewardship behavior. Those who feel that they together are responsible for what is “ours” are likely to take an active role as “stewards” and act in the best interest of what is collectively owned (Hernandez, 2012; Pierce et al., 2017). Organizational psychologists have demonstrated that there is a positive relationship between psychological ownership and stewardship behavior. Employees who have a sense of personal ownership of their work or company are more likely, for example, to commit to extra-role behavior (e.g., Van Dyne & Pierce, 2004; Zhu et al., 2015), and a sense of ownership of public natural areas increases the willingness to protect the area and oppose exploitation (Preston & Gelman, 2020). In addition, a sense of collective ownership of an organization was shown to be related to more stewardship behavior for that organization (Henssen et al., 2014), and a sense of collective ownership of a neighborhood was related to higher local participation (Toruńczyk-Ruiz & Martinovic, 2020).

Although in these studies it is argued that the positive association between psychological ownership and stewardship behavior is due to an increased sense of responsibility, to our knowledge, only Peck et al. (2020) empirically examined and confirmed this indirect link. However, they did so in a context where there is no clearly identified ingroup that can claim collective ownership of the territory (a public park), making it unlikely that collective ownership feelings were involved. In sum, we hypothesize that:

**Hypothesis 2 (H2):** Collective psychological ownership of a territory increases the intention to engage in stewardship behavior indirectly via higher perceived group responsibility.

For reasons of conceptual replication, the two hypotheses were tested cross-sectionally in relation to the country (Study 1) and the neighborhood (Study 2), and experimentally in relation to a local park (Studies 3 and 4), and using slightly different measures and experimental manipulations. These three territories are all targets of collective ownership because, even though individuals might have a sense of personal attachment and belonging to a country, neighborhood, or local park, in general, they will not have a sense of personally owning it.

## Study 1^1^

### Sample

We surveyed a total sample of *N* = 617 Dutch natives via research agency Kantar,^2^ which maintains an online panel. The survey was part of a bigger data collection and the agency stopped collecting data when they reached a sample of approximately 600 participants. The response rate was 42%, which is similar to other survey research in the Netherlands (Stoop, 2005). All participants were 18 years or older (18–92, *M* = 50.57, *SD* = 18.11) and both of their parents had an ethnic Dutch background. The sample was diverse and with weights applied, the sample was representative for the Dutch population in terms of gender, age, and education level. All relevant measures and exclusions are reported for all studies. The materials, data, and code can be found here: https://osf.io/yha3u/.

### Measures

#### Collective psychological ownership

The main independent variable was adapted from previous research on country ownership (Nijs, Martinovic, et al., 2021), which in turn was based on a measure of collective psychological ownership in organizations (Pierce et al., 2017). Three items (7-point scales used for all measures) were used: for example, “I think this country is owned by us, the Dutch”; α = .89 (see Supplemental Appendix B for an overview of all items used in the four studies).

#### Exclusive determination right

Based on the previous research (Nijs, Martinovic, et al., 2021), three items were used: for example, whether Dutch people “have the exclusive right to determine matters that concern The Netherlands” (α = .94).

#### Group responsibility

Three items were designed for the purpose of this research (e.g., “We the Dutch are responsible for The Netherlands”; α = .85).

#### Exclusion of outsiders

We assessed exclusion of outsiders with three items preceded by the general question: “On a scale from 0% (definitely not) to 100% (definitely), what are the chances that you would do the following now or in the future?” A sample item was “vote for a political party that is committed to reducing immigration in The Netherlands” (1 = 0%, 11 = 100%; α = .76).^3^

#### Stewardship behavior

To measure stewardship behavior, we posed the following general question: “On a scale from 0% (definitely not) to 100% (definitely), what are the chances that in the future you will support a charity (by volunteering or donating money) that is committed to . . ..” Four items followed, such as “. . . maintaining and preserving Dutch natural landscapes” (1 = 0%, 11 = 100%; α = .89).

#### Control variables

To examine whether the hypothesized associations existed above-and-beyond other relevant constructs, we controlled for group identification, place attachment, political orientation, national sovereignty, and philanthropy. Collective psychological ownership is based on self-categorization and the related sense of “us” but differs conceptually and empirically from ingroup identification as the feeling of attachment to the ingroup (Brylka et al., 2015; Nijs, Martinovic, et al., 2021; Pierce & Jussila, 2011; Storz et al., 2020). We measured group identification with three items (e.g., “I strongly feel Dutch”; α = .85).

Place attachment concerns a positive affective bond between an individual and a place (Scannell & Gifford, 2010). While collective psychological ownership concerns a sense that “the place belongs to us,” place attachment concerns a sense that “I belong to the place” (Storz et al., 2020). It was measured with three items (α = .82), such as “If I have been outside the country for a while, I am always happy to come back” (Hernández et al., 2007).

We also controlled for political orientation. Left-wing individuals generally endorse equality and change, while right-wing individuals endorse tradition and conformity (Jost, 2006). Political orientation has been previously linked to both exclusion of immigrants (Pettigrew et al., 2007) and stewardship behaviors, such as volunteering and donating to charity (Brooks, 2006; Clerkin et al., 2009). Participants were asked to place themselves on a 7-point scale (1 = *strongly left wing*, 4 = *middle*, 7 = *strongly right wing*) (Jost, 2006).

Sovereignty refers to a political principle about the state’s supreme authority to rule without interference from outside and can, next to collective psychological ownership, account for “why we get to decide about this country” (Ripstein, 2017). It has been found to be an empirically distinct construct from collective psychological ownership (Nijs, Martinovic, et al., 2021) and was measured with three items (e.g., “a country is sovereign and international organizations should not interfere with national regulations”; α = .87).

We measured philanthropy based on a scale designed to capture “the attitude of personal responsibility to the public good” (Schuyt et al., 2004, p. 4). Philanthropy can be an alternative reason to engage in stewardship behavior, next to collective psychological ownership. We used three items (e.g., “We have to make this world a better place for the next generation”; α = .74).

We also controlled for gender (0 = *men*, 1 = *women*), age (in years), and education level (1 = *no primary education*, 9 = *doctorate*). These characteristics have been found to relate to both attitudes toward immigrants (Hernes & Knudsen, 1992) and stewardship behavior (Kreutzwiser et al., 2011) and can also be expected to relate to collective psychological ownership. By controlling for them, we rule out the possibility that they confound the relationships we were interested in.

### Results

#### Measurement model

We performed confirmatory factor analysis in Mplus software (version 8.3) to test whether all nine multi-item variables reflected separate latent constructs. To account for non-normal distributions of endogenous variables, we employed maximum likelihood estimations with robust standard errors (MLR) in all subsequent analyses of all four studies. The expected nine-factor model fitted the model well (comparative fit index [CFI] = .968, root mean square error of approximation [RMSEA] = .035, standardized root mean square residual [SRMR] = .037). Standardized loadings were .67 or higher.

#### Descriptive statistics

Table 1 shows that participants tended to “slightly agree” with the collective psychological ownership items. All constructs that we hypothesized to be related were significantly correlated with each other in the expected positive direction. The strongest correlation was .69 between collective psychological ownership and exclusive determination right, which is similar to previous research (Nijs, Martinovic, et al., 2021). The correlation between collective psychological ownership and group identification was .57 and the correlation between collective psychological ownership and place attachment was .35. A variance inflation factor (VIF) of 2.03 indicates that there are no problems of multicollinearity.^4^

**Table 1.** Descriptive Statistics, Study 1.

	Valid *n*	Range	Mean/ prop.	*SD*	*α*	Correlations			
Variable						2	3	4	5
1. Collective psychological ownership	617	1 to 7	4.87	1.43	.89	.688***	.550***	.493***	.049
2. Exclusive determination right	617	1 to 7	4.31	1.59	.94	1	.445***	.531***	−.015
3. Group responsibility	617	1 to 7	5.78	.90	.85		1	.243***	.245***
4. Exclusion of outsiders	617	1 to 11	3.40	2.44	.76			1	.199***
5. Stewardship behavior	617	1 to 11	6.09	2.60	.89				1
6. Group identification	617	1 to 7	5.48	1.11	.85				
7. Place attachment	617	1 to 7	4.63	1.44	.82				
8. Political orientation	534	1 to 7	4.17	1.41	–				
9. Sovereignty	617	1 to 7	4.85	1.32	.87				
10. Philanthropy	617	1 to 7	5.59	.89	.74				
11. Gender (female)	617	0/1	.50	–	–				
12. Age	617	18 to 92	50.18	18.37	–				
13. Education level	617	1 to 9	4.70	1.75	–				

*Note.* Descriptive statistics were based on manifest mean scores, correlations were between latent variables. α is Cronbach’s alpha. Statistics were based on the weighted data.

* *p* < .05. **p < .01. ***p < .001.

#### Structural model

We specified a path model by regressing exclusion of outsiders and stewardship behavior on determination right, group responsibility, and collective psychological ownership, and by regressing right and responsibility on ownership. All control variables were included as predictors of the mediators and dependent variables. Seventy-two participants who indicated “don’t know/don’t want to answer” on the political orientation variable were coded missing. We used full information maximum likelihood (FIML) to allow for these missing values. We endogenized all exogenous variables, meaning that all variables were allowed to covary.^5^

**Figure 1.**
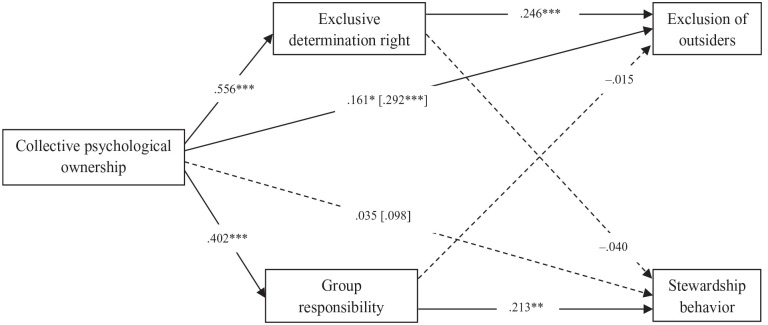
Standardized coefficients of the path model of Study 1. *Note.* Total effects were reported between square brackets. Included control variables were not reported. *p < .05. **p < .01. ***p < .001.

Figure 1 shows that collective psychological ownership of the country was positively related to perceived determination right, which in turn was related to higher intentions to exclude outsiders. This led to a positive and significant indirect effect of ownership on exclusion of outsiders via determination right (β = .136, *SE* = .039, *p* = .001^6^), in line with H1. There was also a significant positive total effect and a positive direct effect of ownership on exclusion of outsiders. There was no significant indirect effect via group responsibility (β = -.006, *SE* = .026, *p* = .822).

Simultaneously, collective psychological ownership was positively related to group responsibility, which in turn was related to higher intentions to engage in stewardship behavior. This led to a positive indirect effect of ownership on stewardship behavior via group responsibility (β = .086, *SE* = .031, *p* = .006), in line with H2. However, there was no significant total effect of ownership on stewardship behavior. There was also no significant direct effect, nor indirect effect via exclusive determination right (β = -.022, *SE* = .040, *p* = .580). All results concerning control variables are shown in Supplemental Appendix D. Note that a model without control variables (see Supplemental Appendix E) showed the same pattern of results, except that exclusive determination right was additionally negatively related to stewardship behavior (β = −.135, *SE* = .065, *p* = .038).

### Discussion

We found that collective psychological ownership of the country went together with both a perceived exclusive determination right and perceived group responsibility. Determination right, in turn, was related to higher intentions to exclude outsiders (i.e., immigrants), while responsibility was related to higher intentions to engage in stewardship behavior. Thus, we found evidence for both hypothesized indirect paths.

## Study 2

Study 1 offered novel insights into the importance of collective psychological ownership for different behavioral intentions. In Study 2, our aim was to conceptually replicate the same model in relation to another relevant and more concrete, everyday shared territory—the neighborhood.

### Sample

Via research agency Motivaction, we recruited a sample of 831 Dutch adults from an online panel called StemPunt. It was part of a bigger data collection and the agency stopped collecting data when they reached a sample of approximately 800 participants. We excluded 47 participants because they did not pass the speeding check leading to a final sample of *N* = 784.^7^ All participants were 18 years or older and all had at least one parent born in the Netherlands. The sample was not representative of the Dutch population as weights were not available. However, the sample was diverse in terms of gender (53% women) age (18-79, *M* = 51.10, *SD* = 16.51), and education level (20% low secondary school or less, 44% high school or vocational training, and 36% [applied] university).

### Measures

We adapted the measures from Study 1 to the neighborhood context (see Supplemental Appendix B). Example items were “I strongly feel like this is our neighborhood” (collective psychological ownership; *r* = .88), “My neighbors and I have the right to determine matters that concern our neighborhood” (exclusive determination right; α = .83), “My neighbors and I are responsible for our neighborhood” (group responsibility; α = .87), “support a local initiative that first offers vacant housing to current neighborhood residents” (exclusion of outsiders; α = .87), and “maintain a flowerbed or garden in your neighborhood” (stewardship behavior; α = .90).

We controlled for group identification, place attachment, gender (0 = *men*, 1 = *women*),^8^ age (in years), education level (1 = *no primary education*, 9 = *doctorate*), and mixed ethnic background status (1 = one parent not born in the Netherlands, 0 = both parents born in the Netherlands). Group identification (e.g., “I identify with other neighbors”) and place attachment (e.g., “When I am gone, I miss my neighborhood”) were also adapted to the neighborhood context. Moreover, we controlled for self-reported characteristics of the participants’ neighborhood and place of residence, as these characteristics might influence all variables in the model. We asked participants to indicate the size of their place of residence: a big city (> 100,000 residents); an average city (50,000-100,000 residents); a small city (< 50,000 residents); a village. The variable was treated as categorical, with village as the reference category. We measured length of residence in the neighborhood in years.^9^ Share of newcomers was measured by asking participants to estimate the percentage (0-100%) of people in the neighborhood who have been living there for less than three years. In addition, we measured social cohesion by asking participants to indicate how often they have a conversation with at least one neighbor (1 = n*ever or barely*, 5 = *every day*) (Dassopoulos & Monnat, 2011).

### Results

We first performed confirmatory factor analyses to test whether all multi-item variables captured separate latent constructs. The expected seven-factor model fitted the data well (CFI = .962, RMSEA = .048, SRMR = .047). Standardized loadings were .69 or higher. Table 2 shows all descriptive statistics. Participants tended to “slightly agree” with the collective psychological ownership items and scored low on the exclusion of outsiders. All constructs that we hypothesized to be related correlated in the expected positive direction. Moreover, collective psychological ownership correlated relatively strongly with both group identification (*r* = .63) and place attachment (*r* = .68). There were no signs of multicollinearity (VIF = 2.01).

**Table 2.** Descriptive Statistics, Study 2.

	Valid *n*	Range	Mean/prop.	*SD*	α	Correlations			
Variable						2	3	4	5
1. Collective psychological ownership	783	1 to 7	4.91	1.46	.88^a^	.390***	.513***	.061	.314***
2. Exclusive determination right	784	1 to 7	4.14	1.24	.83	1	.566***	.233***	.248***
3. Group responsibility	784	1 to 7	4.77	1.17	.87		1	.140**	.558***
4. Exclusion of outsiders	784	1 to 11	2.67	2.14	.87			1	.270***
5. Stewardship behavior	784	1 to 11	5.52	2.66	.90				1
6. Group identification	784	1 to 7	3.40	1.46	.84				
7. Place attachment	784	1 to 7	4.28	1.52	.84				
8. Gender (female)	783	0/1	.53	–	–				
9. Age	784	19 to 79	51.10	16.51	–				
10. Education level	783	1 to 9	5.21	1.89	–				
11. Mixed ethnic background	784	0/1	.06	–	–				
12. Place of residence size	784								
Big city	784	0/1	.25	–	–				
Average city	784	0/1	.21	–	–				
Small city	784	0/1	.16	–	–				
Village (reference category)	784	0/1	.38	–	–				
13. Length of neighborhood residence	781	0 to 70	18.04	14.60	–				
14. Share of newcomers	783	0 to 100	23.83	21.56	–				
15. Social cohesion	784	1 to 5	3.49	1.04	–				

*Note.* Descriptive statistics were based on manifest mean scores, correlations were between latent variables. *α* is Cronbach’s alpha.

a Correlation between two items, instead of Cronbach’s alpha.

* *p* < .05. **p < .01. ***p < .001.

**Figure 2.**
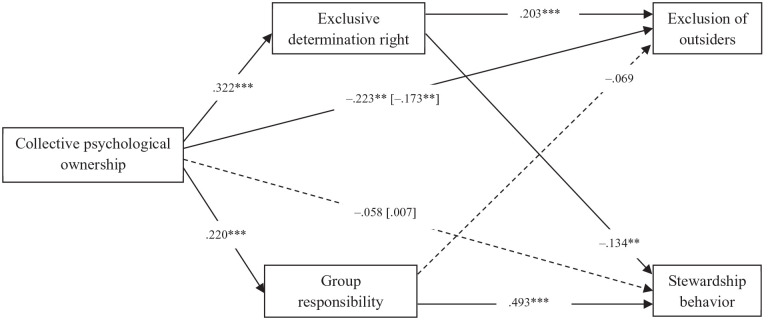
Standardized coefficients of the path model of Study 2. *Note.* Total effects were reported between square brackets. Included control variables were not reported. *p < .05. **p < .01. ***p < .001.

We specified the same path model as in Study 1 with all exogenous variables being endogenized.^10^ Collective psychological ownership of the neighborhood was positively related to exclusive determination right, which in turn was related to higher intentions to exclude outsiders (Figure 2).^11^ Moreover, collective psychological ownership of the neighborhood was also positively related to perceived responsibility, which in turn was associated with more intentions to engage in stewardship behavior. Both the positive indirect effect of ownership on exclusion of outsiders via determination right (β = .066, *SE* = .024, *p* = .006) and on stewardship behavior via perceived responsibility (β = .108, *SE* = .032, *p* = .001) were significant, again providing evidence for H1 and H2, respectively. As in Study 1, there was no significant total effect nor direct effect of ownership on stewardship behavior.

However, there were also some findings that we did not anticipate. Concerning exclusion of outsiders, we found negative direct and total effects of collective psychological ownership. Note that the negative total effect only appeared when controlling for group identification (see Supplemental Appendix G), which is significantly positively related to exclusion of outsiders (β = .445, *SE* = .072, *p* < .001). When no control variables were taken into account, the direct and total effects of ownership on exclusion of outsiders were not significant, in line with the non-significant bivariate correlation shown in Table 2. With regard to stewardship behavior, we found that stronger perceived determination right was associated with less stewardship behavior and there was a significant negative indirect effect of ownership on stewardship behavior via determination right (β = -.043, *SE* = .016, *p* = .009). We found the same pattern of associations when no control variables were taken into account (see Supplemental Appendix H), except for a positive total effect of ownership on stewardship behavior (β = .312, *SE* = .037, *p* < .001).

### Discussion

Study 2 conceptually replicated the findings of Study 1. Similar to country collective ownership, neighborhood collective ownership was accompanied by a perceived exclusive determination right which in turn was related to higher intentions to exclude outsiders (i.e., those not living in the neighborhood). Simultaneously, neighborhood ownership was associated with group responsibility, and indirectly to more intentions to engage in stewardship behavior.

## Study 3

The findings discussed were based on cross-sectional data, which prevents conclusions about the direction of influence. Therefore, in Study 3, we used a survey-embedded experiment to examine the effect of collective psychological ownership on rights and responsibilities, and subsequently on behavioral intentions. The same path model was tested as in the previous studies, but in relation to yet another shared territory, namely, an imaginary local park.

### Sample and Procedure

We recruited 384 adult Dutch natives via research agency Kantar. Assuming a small to moderate effect size (Cohen’s *d* = .3), a priori power calculations (aiming for a power of .80 at the alpha .05 level) suggested a required sample size of 352 (176 participants per experimental condition). The experiment was pre-registered on the Open Science Framework.^12^ The sample was diverse in terms of gender (52% women), age (18-93, *M* = 51.07, *SD* = 16.89), and education level (23% low secondary school or less, 42% high school or vocational training, and 35% [applied] university). With weights applied, the sample was representative for the Dutch population in terms of gender, age, and education level.

In recent studies, researchers have manipulated individual psychological ownership by asking participants to think of a (nick)name for the target of ownership, by showing signs with personal possessive pronouns, by investing time and energy in it, or using it (Peck et al., 2020; Preston & Gelman, 2020). For triggering a sense of collective ownership, we presented similar features that people generally use to infer and claim ownership (Verkuyten & Martinovic, 2017). Participants were randomly assigned to either a collective ownership condition (*N* = 205) or a control condition (*N* = 179). In both conditions, we asked participants to imagine that there was a small park in their street. In the ownership condition they read that not much happened in the park before, and that “you and your neighbors” have renewed it and put a picknick table there. It was explained that “you and your neighbors” go there a lot, really feel like it is “your park” (in Dutch the collective possessive pronoun “*jullie*” was used), and even gave it the name “our green park.” In the control condition, we only mentioned that “the people in your street” hardly use the park. In both conditions, a photo shows the same little park with a picknick table. The design was based upon an experiment used to manipulate collective ownership threat (Nijs, Verkuyten, & Martinovic, 2021, Study 1). See the exact wording of the experiment in Supplemental Appendix I.

### Measures

After the manipulation, participants answered two items (e.g., “If I think about the park, I really feel that it is owned by us, neighbors,” *r* = .78) to check whether the ownership condition increased collective psychological ownership of the local park, compared to the control condition. All measures were adapted to the context of the imaginary local park (Supplemental Appendix B). Subsequently, items measuring exclusive determination right (e.g., “It is up to me and my neighbors to determine what happens in the park,” α = .89) and group responsibility (e.g., “My neighbors and I are responsible for the park,” α = .92) were presented in a random order. This was followed by the items on the exclusion of outsiders and stewardship behavior which were also presented in a random order. Exclusion of newcomers was measured as follows:

Now imagine that the park is recently used more and more by people who do not live in your street. On a scale from 0% (definitely not) to 100% (definitely), what are the chances that you would do the following?

A sample item was “Place a sign that reads ‘for local residents’” (1 = *0%*, 11 = *100%*; α = .88). Stewardship behavior was measured by asking about the chance that one would, for example, “clean up litter in the park” (α = .89).

### Results

Confirmatory factor analyses indicated that the expected four-factor model fitted the data well (CFI = .947, RMSEA = .072, SRMR = .057) with standardized loadings > .74. Table 3 shows mean scores and standard deviations of the variables by experimental condition. Confirming that the manipulation was successful, participants scored higher on the collective psychological ownership items in the ownership condition compared to the control condition. Moreover, participants in the ownership condition also scored significantly higher on all the variables, compared to participants in the control condition.

**Table 3.** Descriptive Statistics, Study 3.

	Range	α	Ownership condition		Control condition		*t*
Variable			*M*	*SD*	*M*	*SD*	
Collective psychological ownership (manipulation check)	1 to 7	.78^a^	5.25	1.16	4.38	1.47	6.52***
Exclusive determination right	1 to 7	.89	4.25	1.39	3.81	1.41	3.04**
Group responsibility	1 to 7	.92	5.08	1.12	3.64	1.44	11.02***
Exclusion of outsiders	1 to 11	.88	3.74	2.48	2.89	2.14	3.55***
Stewardship behavior	1 to 11	.89	6.61	2.37	4.66	2.59	7.70***

*Note.* Descriptive statistics were based on manifest mean scores. α is Cronbach’s alpha. *t* is the *t*-statistic of difference in mean across the two conditions. Statistics were based on the weighted data.

a Correlation between two items, instead of Cronbach’s alpha.

* *p* < .05. **p < .01. ***p < .001.

**Figure 3.**
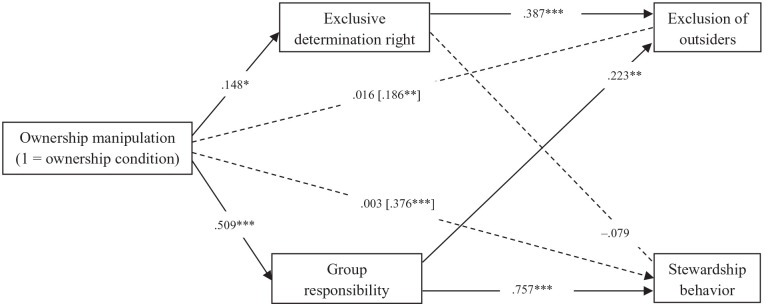
Standardized coefficients of the path model of Study 3. *Note.* Total effects were reported between square brackets. *p < .05. **p < .01. ***p < .001.

We specified a path model by regressing exclusion of outsiders and stewardship behavior on exclusive determination right, group responsibility, and the ownership manipulation (1 = ownership condition; 0 = control condition), and by regressing right and responsibility on the ownership manipulation. Similar to the first two studies, Figure 3 shows that participants in the ownership condition had stronger perceptions of exclusive determination right, which in turn was related to higher intentions to exclude outsiders. This led to a significant positive indirect effect of the ownership condition on exclusion of outsiders via perceived determination right (β = .057, *SE* = .025, *p* = .021), confirming H1. The ownership manipulation also led to stronger perceptions of group responsibility, which in turn was associated with higher intentions to perform stewardship behavior. As hypothesized (H2), we have detected a significant positive indirect effect of the ownership manipulation on stewardship behavior via perceived responsibility (β = .385, *SE* = .049, *p* < .001).

One unexpected finding was that exclusion of outsiders was increased not only by stronger perceptions of determination right, but also by stronger perceptions of group responsibility, which led to a significant indirect effect of the ownership manipulation on exclusion of outsiders via responsibility (β = .113, *SE* = .039, *p* = .004). In addition, unlike the previous two studies, we found a significant positive total effect of the ownership manipulation on stewardship behavior. See all results in Supplemental Appendix J.

### Discussion

In Study 3, we experimentally showed that increasing a sense of collective ownership of an imaginary local park causes a higher perception of exclusive determination right, and indirectly, stronger intentions to exclude outsiders. At the same time, increasing collective ownership of the park also led to higher perceived group responsibility, and indirectly, to stronger intentions to engage in stewardship behavior.

## Study 4

In Study 4, we used the same experimental design and measures as in Study 3 but with two relevant changes.^13^ First, people use all sorts of features to infer ownership, and in Study 3, we successfully elicited a sense of collective ownership with a short narrative in which the features of investment and naming, and also liking and usage were mentioned. In the control condition it was mentioned that neighbors hardly make use of the park. Liking and frequency of usage, however, do not have to indicate ownership and might have been (partly) responsible for the findings. Therefore, in Study 4, the manipulation focused more clearly on ownership features by stating that the neighbors (vs. the municipality) have invested in the park. Second, the formulation of some of the items in Study 3 might (partly) have assessed perceived individual rights and responsibilities and behavioral intentions (“the chances that you would do the following”). Therefore, in Study 4, we improved on the measures so that all items were clearly and explicitly formulated at the collective level.

### Sample and Procedure

The experiment was pre-registered on the Open Science Framework^14^ and the procedure was exactly the same as in Study 3. We assumed a small to moderate effect size (Cohen’s *d* = .3) and aimed for a power of .80 at the alpha .05 level, as in Study 3. Therefore, we again required a minimum sample size of 352. The experiment was part of a larger data collection and in the end, 502 adult participants filled in the questionnaire. The sample was diverse in terms of gender (53% women), age (18-95, *M* = 49.66, *SD* = 17.18), and education level (29% low secondary school or less, 42% high school or vocational training, and 29% [applied] university). Weights were applied to make the sample representative for the Dutch population in terms of gender, age, and education level. As in Study 3, participants were randomly assigned to either a collective ownership condition (*N* = 230) or a control condition (*N* = 272). Participants were asked in both conditions to imagine that there was a small park in their street. The manipulation was the same as in Study 3 but without mentioning usage and liking and with adding investment by the neighbors or the municipality. In the ownership condition, participants read that the small park used to be just a piece of land where nothing happened, but that the municipality has given it to the neighborhood. It was also mentioned that “you and your neighbors” have tidied it up and put up a picnic table. In the control condition, participants also read that it was just a piece of land where nothing happened, but that the municipality had tidied it up and put up a picnic table (see for the exact wording, Supplemental Appendix K).

### Measures

The manipulation check was the same as in Study 3. The measures for exclusive determination right and group responsibility were also the same, except that only collective nouns and pronouns were used (e.g., “we neighbors” instead of “my neighbors and I”; Supplemental Appendix B). Furthermore, the measures for “exclusion of outsiders” and “stewardship behavior” explicitly focused on intentions to take collective actions. For example, we measured exclusion of outsiders by asking “what are the chances that you as local residents together would do the following?,” instead of “what are the chances that you would do the following?” (Study 3).

### Results

Confirmatory factor analyses again showed that the expected four-factor model fitted the data well (CFI = .985, RMSEA = .043, SRMR = .037), with standardized loadings > .78. Table 4 shows that participants scored higher in the ownership condition compared to the control condition on the collective psychological ownership items, indicating that the manipulation was successful. The average scores for the other measures were also significantly higher in the ownership condition.

**Table 4.** Descriptive Statistics, Study 4.

	Range	α	Ownership condition		Control condition		*t*
Variable			*M*	*SD*	*M*	*SD*	
Collective psychological ownership (manipulation check)	1 to 7	.84^a^	5.38	1.17	4.86	1.29	4.75***
Exclusive determination right	1 to 7	.91	4.48	1.33	3.77	1.47	5.64***
Group responsibility	1 to 7	.94	5.05	1.36	4.10	1.45	7.58***
Exclusion of outsiders	1 to 11	.90	4.10	2.34	3.35	2.29	3.61***
Stewardship behavior	1 to 11	.92	6.53	2.61	5.03	2.56	6.51***

*Note.* Descriptive statistics were based on manifest mean scores. α is Cronbach’s alpha. *t* is the *t*-statistic of difference in mean across the two conditions. Statistics were based on the weighted data.

a Correlation between two items, instead of Cronbach’s alpha.

* *p* < .05. **p < .01. ***p < .001.

**Figure 4.**
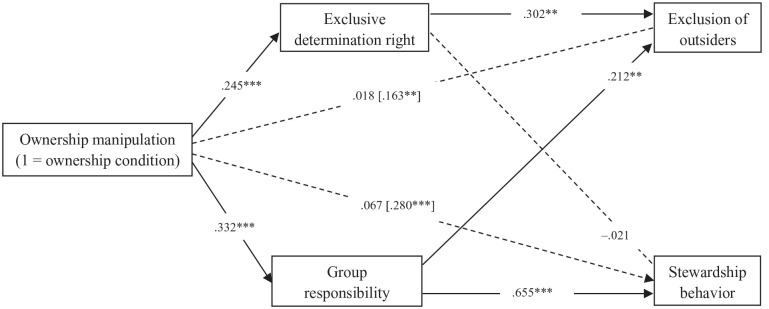
Standardized coefficients of the path model. *Note.* Total effects were reported between square brackets. *p < .05. **p < .01. ***p < .001.

As shown in Figure 4, the results show a very similar pattern as in Study 3 (Figure 3). In line with H1 and H2, respectively, we again found a significant positive indirect effect of the collective ownership manipulation on exclusion of outsiders via perceived determination right (β = .074, *SE* = .028, *p* = .009), and a significant positive indirect effect of the collective ownership manipulation on stewardship behavior via perceived responsibility (β = .218, *SE* = .034, *p* < .001). Also similar to Study 3 and unexpected, exclusion of outsiders was increased not only by exclusive determination right but also by group responsibility, as seen from a positive and significant indirect effect of the ownership manipulation on exclusion of outsiders via responsibility (β = .070, *SE* = .029, *p* = .014). See all results in Supplemental Appendix L.

## General Discussion

Following self-categorization theory (Turner et al., 1987) and an intergroup perspective, we focused on collective psychological ownership (“ours”) of shared territories (country, neighborhood, and local park) for which a sense of individual ownership (“mine”) is not very likely. The findings demonstrate that collective psychological ownership has different aspects, and by examining both perceived rights and responsibilities and exclusionary and prosocial behavioral implications, we offer a comprehensive picture of the diverse consequences of collective psychological ownership. In addition, by focusing on shared territories as the collective targets of ownership, we illustrated the importance of taking the physical environment into account in social psychology, and in intergroup research in particular (Meagher, 2020).

We confirmed the intuitive link between collective psychological ownership and the exclusive determination right (Katz, 2008; Merrill, 1998) that was previously found in relation to the country (Nijs, Martinovic, et al., 2021), but not yet in relation to other shared territories. Exclusive determination right, in turn, predicted the behavioral tendency to exclude outsiders. At the same time, we found that collective psychological ownership was also accompanied by higher group responsibility (Verkuyten & Martinovic, 2017). In organizational psychology, psychological ownership has been linked to personal responsibility (e.g., Kamleitner & Rabinovich, 2010; Pierce & Jussila, 2011), but the association between collective psychological ownership and a sense of group responsibility has not been considered before. Group responsibility, in turn, predicted the intention to engage in stewardship behavior. Thus, those who had a more pronounced sense of “this is ours” felt more strongly that their group was responsible for what they collectively owned and were therefore more willing to take care of “what is ours.”

To our knowledge, this is a first set of studies to show that a sense of collective ownership of territories is accompanied by both perceived group rights and group responsibilities. Importantly, our expectations were confirmed using both cross-sectional and experimental designs. Whereas the intention to exclude outsiders and stewardship behavior can be outcomes of collective psychological ownership, people might also justify their intentions and behaviors with collective ownership beliefs. In the experimental Studies 3 and 4, however, we found that collective psychological ownership causes higher perceived exclusive determination right and also stronger group responsibility.

Our main finding that collective psychological ownership has different implications was conceptually replicated across three shared territories at different levels of abstraction, using different methods, and using partly different phrasings in the measures and manipulations. By repeatedly testing the same theoretical mechanisms in varying ways, we increased confidence in the generalizability of our observations and the theoretical propositions underlying them (Crandall & Sherman, 2016). Therefore, we believe that our conclusions are not limited to the territories examined but can be expected to be found in relation to different territorial targets of collective ownership, such as regions or cities, or shared housing or offices. However, the findings also tell us something specific about ownership of the country, the neighborhood, and a local park specifically. Concerning the country, Study 1 showed that a sense that “this country is ours” is associated not only with the exclusion of immigrants, but also with more group responsibility and investment in the country. Collective ownership rhetoric might therefore not only be used by politicians to argue for anti-immigration measures, but also for the importance of citizens’ civic engagement and involvement in taking care of their country.

Concerning neighborhoods as the target of collective ownership feelings, we showed that ownership relates to group responsibility and indirectly to stewardship behavior. Many urban policies in various countries focus on encouraging local residents to take responsibility for and to be involved in “their” neighborhood as this contributes to local safety, livability, and social cohesion (Dekker, 2007). Our findings suggest that a sense of collective ownership can help achieve this goal. Moreover, bivariate correlations showed that neighborhood collective ownership did not relate to intentions to exclude outsiders and total effects even showed that ownership was related to less intentions to exclude outsiders when controlling for group identification. This was an unexpected finding. One possible explanation is that in the neighborhood, a sense of “we” was strongly related to more exclusion of outgroups and confounded the exclusionary side of ownership. Policy makers and community workers might conclude that we should try to increase collective psychological ownership of the neighborhood as it stimulates shared responsibility—and in turn stewardship behavior—and does not increase intentions to exclude outsiders. However, our research did not distinguish between native newcomers moving to the neighborhood from other regions of the country and international migrants. Toruńczyk-Ruiz and Martinovic (2020) found that neighborhood ownership related to more openness to newcomers from within the country but to less openness to international migrants. We did not explicitly mention the outsiders’ backgrounds in our measures related to the neighborhood, but it might be that participants thought of newcomers from within the Netherlands. One other unexpected finding was that those who felt that they and their neighbors had the right to determine what happens to their neighborhood were less likely to engage in stewardship behavior. It might be that they felt that they properly controlled their neighborhood, and therefore, did not find it necessary to improve the neighborhood with any of the stewardship behaviors.

Experimental Studies 3 and 4 examined the role of collective psychological ownership of a local park. Local residents do not always recognize themselves as responsible for areas that are public property (Moskell & Allred, 2013). Facilitating residents to put time and effort into a local area to make it “their own” can help to foster responsibility and stewardship behavior, which can strengthen a local community and can improve the neighborhood. However, our study shows that a sense of ownership of a local setting can also lead to the intention to exclude outsiders. These results are in line with geography research that in addition to prosocial consequence, points at the exclusionary consequences of shared ownership of community gardens (Spierings et al., 2018; Van Holstein, 2016). Unexpectedly, the intention to exclude outsiders from the local park was predicted not only by higher perceived determination right, but also by higher group responsibility. Residents might assume that people from outside the neighborhood will not take proper care of the park and therefore feel like taking responsibility by sending them away. This interpretation should be examined in future research.

## Conclusion

A sense of ownership is a key aspect of social life that self-evidently structures social relations between people (Verkuyten & Martinovic, 2017). However, attention to the critical role of ownership and also to the importance of the physical environment and territory has been limited in the social psychology literature (Meagher, 2020). Our research demonstrates that perceived collective territorial ownership can play an important role in social attitudes and behaviors. We have shown that the feeling that a shared place is “ours” has different aspects and these aspects can have distinct behavioral implications in different contexts. Collective psychological ownership of territories is not only an obstacle to peaceful intergroup relations but can also stimulate people to invest into “their” place. A sense of collective ownership is a central feature of the social world and therefore an important social psychological topic that deserves more systematic research: not only in relation to territories but also in relation to, for example, artifacts, social representations, and forms of cultural appropriation.

## Supplemental Material

sj-docx-1-psp-10.1177_01461672221129757 – Supplemental material for The Two Routes of Collective Psychological Ownership: Rights and Responsibilities Explain Intentions to Exclude Outsiders and Engage in Stewardship Behavior Click here for additional data file. Supplemental material, sj-docx-1-psp-10.1177_01461672221129757 for The Two Routes of Collective Psychological Ownership: Rights and Responsibilities Explain Intentions to Exclude Outsiders and Engage in Stewardship Behavior by Tom Nijs, Borja Martinovic and Maykel Verkuyten in Personality and Social Psychology Bulletin

## Notes

1. We piloted the design of Study 1. Results of this pilot study were not substantially different, and we have included these in Appendix A of the supplementary material.

2. In total, 1,001 participants were surveyed. Respondents were assigned to one of two versions of the questionnaire. Only 617 of the participants received a version that was about country ownership. The other 384 received the experimental manipulation used for Study 3. The participants included in Study 1 did not receive a manipulation.

3. For balance purposes, the matrix including the items measuring exclusion of outsiders also included three items to measure inclusion of outsiders. These items were not used to make the measure exclusion of outsiders consistent across the studies. When we did include the three reversed inclusion of outsiders items, the measurement model fitted the data worse than when they were not included (CFI = .903, RMSEA = .059, SRMR = .082), but the structural model did not substantially change.

4. In all studies, VIF was calculated using manifest mean scores.

5. When endogenizing the dichotomous variable gender, Mplus warned that standard errors may not be trustworthy. However, results did not substantially change when gender was not endogenized.

6. See Supplemental Appendix C for the confidence intervals in all studies.

7. Participants who were faster than one-third of the median duration of the full survey were considered speeders (Miller et al., 2020).

8. One participant who indicated “other” as their gender was coded missing, for statistical reasons only.

9. Two participants were coded missing as they indicated 100 years as their length of neighborhood residence, while aged 39 and 51.

10. Endogenizing the dichotomous variables (i.e., gender, mixed ethnic background, and place of residence size) caused the same warning in Mplus as in Study 1, but the results did not substantially change when the dichotomous variables were not endogenized.

11. As part of an experimental manipulation that was not used in this study, a random half of the participants were asked to do a short task related to their neighborhood. To check whether this manipulation influenced our results, we included the versions as predictors of all endogenous variables in Supplemental Appendix F. This showed that our results were not influenced by the experimental manipulations.

12. See https://osf.io/e4bg6. As indicated in the pre-registration, we also explored whether the experiment influenced participation in the decision-making process of the park (e.g., “Participate in a meeting of the municipality about the future of the park”; α = .90). We found that it was heightened by the manipulation (total effect: β = .197, *SE* = .056, *p* < .001), which was explained by more responsibility (indirect effect: β = .190, *SE* = .051, *p* < .001) and not by determination right (indirect effect: β = -.018, *SE* = .014, *p* = .199). We decided to not add it as another dependent variable in the main model, as we do not consider it a measure of stewardship behavior. However, adding it to the model does not change our results.

13. We like to thank a reviewer and the editor for suggesting these changes in trying to replicate the findings of Study 3.

14. See https://osf.io/sqa3r.
