# Supplementary material for: The Two Routes of Collective Psychological Ownership: Rights and Responsibilities Explain Intentions to Exclude Outsiders and Engage in Stewardship Behavior
Source: Pers Soc Psychol Bull. 2022 Oct 26;50(2):270–84. doi: 10.1177/01461672221129757 (PMC10860364; doi:10.1177/01461672221129757)
Supplement: sj-docx-1-psp-10.1177_01461672221129757 – Supplemental material for The Two Routes of Collective Psychological Ownership: Rights and Responsibilities Explain Intentions to Exclude Outsiders and Engage in Stewardship Behavior [file sj-docx-1-psp-10.1177_01461672221129757.docx]

**Supplementary material**

The two routes of collective psychological ownership: Rights and responsibilities explain intentions to exclude outsiders and engage in stewardship behavior

**Appendix A.** Pilot Study

**Sample**

To pilot the research design of Study 1, we recruited a sample of 425 adult Dutch natives from the online panel used in Study 2. None of these participants also took part in Study 2. All participants were eighteen years or older and they and both of their parents were born in The Netherlands. We excluded nine participants who did not pass the speeding check,^[[1]](#footnote-1)^ leading to a final sample of *N* = 416. The sample was diverse in terms of gender (50% women), age (19-80, *M* = 48.10, *SD* = 16.17), and education level (24% low secondary school or less, 42% high school or vocational training, and 34% [applied] university). There was no weight available.

**Measures**

The measures were similar to those in Study 1 (see Table A-1). We controlled for group identification, political orientation, gender (0 = *men*, 1 = *women*), age (in years), and education level (1 = *primary education*, 8 = *doctorate*). We measured group identification with one item: ‘to what extent do you feel Dutch?’ (1 = *not at all*, 7 = *very strongly*).

| **Table A-1.** Translations of the exact wording of the variables used in the Pilot Study. | | |
| --- | --- | --- |
| Collective psychological ownership | Intro | Think about the house, automobile, workspace, or some other item that you own or co-own with someone, and the feelings associated with the statement "THIS IS MINE/OURS!"  The following statements refer to the feeling of owning the country The Netherlands together with other Dutch people. Please indicate to what extent you disagree or agree with the following statements. |
|  | 1 | I think this country is owned by us, the Dutch |
|  | 2 | I feel that this country belongs to us, the Dutch |
|  | 3 | I have the feeling that we, the Dutch, own this country together |
|  | | |
| Exclusive determination right | Intro | To what extent do you agree or disagree that Dutch people ... |
|  | 1 | … have the exclusive right to determine matters that concern The Netherlands |
|  | 2 | … have the exclusive right to determine who will be allowed in The Netherlands |
|  | 3 | … have the exclusive right to determine what happens to The Netherlands in the future |
|  | | |
| Group responsibility | Intro | To what extent do you agree or disagree that the Dutch ... |
|  | 1 | … have the responsibility to make sure that The Netherlands is a nice country to live in |
|  | 2 | … have the duty to invest in solving problems in The Netherlands |
|  | 3 | … have the responsibility to leave The Netherlands in a good state for future generations |
|  | | |
| Exclusion of outsiders | Intro | On a scale from 0% (definitely not) to 100% (definitely), what are the chances that you would do the following now or in the future? |
|  | 1 | Vote for a political party that is committed to reducing immigration in The Netherlands |
|  | 2 | Sign a petition against new immigrants coming to The Netherlands |
|  | 3 | Participate in a protest against growing immigration in The Netherlands |
|  | | |
| Stewardship behavior | Intro | On a scale from 0% (definitely not) to 100% (definitely), what are the chances that you would do the following now or in the future? |
|  | 1 | Voluntary participate in an initiative to clean up litter |
|  | 2 | Participate in a protest against the arrival of a polluting company to The Netherlands |
|  | 3 | Support charity to preserve Dutch natural landscapes |

**Results**

Confirmatory factor analyses showed that the expected five-factor model had an acceptable fit after freeing the covariance of the residuals of the items ‘participate in a protest against growing immigration in The Netherlands’ measuring exclusion of outsiders, and ‘participate in a protest against the arrival of a polluting company to The Netherlands’ measuring stewardship behavior (CFI = .950, RMSEA = .069, SRMR = .053). This cross-factor covariance was added in the subsequent analyses. Standardized loadings were .54 or higher. Table A-2 shows all descriptive statistics.

| **Table A-2.** Descriptive statistics, Pilot Study | | | | | | | | | |  |
| --- | --- | --- | --- | --- | --- | --- | --- | --- | --- | --- |
|  | *Valid n* | *Range* | *Mean/ prop.* | *SD* | *α* | *Correlations* | | | | |
|  |  |  |  |  |  | 2. | 3. | 4. | 5. | |
| 1. Collective psychological ownership | 416 | 1-7 | 4.80 | 1.52 | .88 | .649*** | .277*** | .493*** | –.082 | |
| 2. Exclusive determination right | 416 | 1-7 | 4.58 | 1.65 | .93 | 1 | .236*** | .601*** | –.132 | |
| 3. Group responsibility | 416 | 1-7 | 5.98 | .91 | .89 |  | 1 | .132* | .271*** | |
| 4. Exclusion of outsiders | 416 | 1-11 | 4.63 | 3.16 | .89 |  |  | 1 | –.116 | |
| 5. Stewardship behavior | 416 | 1-11 | 6.42 | 2.34 | .70 |  |  |  | 1 | |
| 6. Group identification | 416 | 1-7 | 6.30 | .94 | - |  |  |  |  | |
| 7. Political orientation | 416 | 1-7 | 4.05 | 1.35 | - |  |  |  |  | |
| 8. Gender (female) | 415 | 0/1 | .50 | - | - |  |  |  |  | |
| 9. Age | 416 | 19-80 | 48.10 | 16.17 | - |  |  |  |  | |
| 10. Education level | 416 | 1-8 | 4.12 | 1.89 | - |  |  |  |  | |
| *Note:* Descriptive statistics were based on manifest mean scores, correlations were between latent variables. *α* is Cronbach’s alpha*.* **p* < .05, ***p <* .01; ****p* < .001. | | | | | | | | | |  |

We specified the same mediation model as in Study 1. Figure A-1 shows that the results did not substantially differ from the results in Study 1. See all results concerning control variables in Table A-3.

Collective psychological ownership

Exclusive determination right

Group responsibility

Exclusion of outsiders

Stewardship behavior

.577***

.152* [.357***]

–.006 [.008]

.268***

.359***

–.008

–.119

.308***

**Figure A-1.** Standardized coefficients of the path model of the Pilot Study. Total effects were reported between square brackets. Included control variables were not reported. **p* < .05; ***p* < .01 ****p* < .001.

| **Table A-3.** Standardized regression coefficients for the mediation model, Pilot Study | | | | |
| --- | --- | --- | --- | --- |
|  | Exclusive determination right | Group responsibility | Exclusion of outsiders | Stewardship behavior |
| *Direct effects* |  |  |  |  |
| Collective psychological ownership | .577 (.053)*** | .268 (.063)*** | .152 (.060)* | -.006 (.087) |
| Exclusive determination right |  |  | .359 (.065)*** | -.119 (.086) |
| Group responsibility |  |  | -.008 (.044) | .308 (.071)*** |
|  |  |  |  |  |
| Group identification | -.059 (.041) | .035 (.054) | -.052 (.041) | -.120 (.056)* |
| Political orientation | .222 (.049)*** | -.045 (.052) | .322 (.055)*** | -.182 (.061)** |
| Gender (female) | .045 (.039) | -.047 (.053) | -.017 (.042) | .070 (.054) |
| Age | .032 (.042) | .057 (.054) | -.088 (.043)* | .290 (.061)*** |
| Education | -.094 (.043)* | .032 (.053) | -.135 (.044)** | .119 (.064) |
|  |  |  |  |  |
| *Indirect effects* |  |  |  |  |
| Collective psychological ownership > exclusive determination right |  |  | .207 (.041)*** | -.069 (.051) |
| Collective psychological ownership > group responsibility |  |  | -.002 (.012) | .083 (.029)** |
|  |  |  |  |  |
| *Total effects* |  |  |  |  |
| Collective psychological ownership |  |  | .357 (.052)*** | .008 (.072) |
|  |  |  |  |  |
| R^2^ | .479 | .085 | .484 | .235 |
| N | 416 |  |  |  |
| * *p <* .05; ** *p <* .01; *** *p* < .001. | | | | |

| **Appendix B.** Translations of the exact wording of all multi-item variables used in the four studies | | | |
| --- | --- | --- | --- |
|  |  | Study 1 | Study 2 |
| Collective psychological ownership | Intro | Think about the house, automobile, workspace, or some other item that you own or co-own with someone, and the feelings associated with the statement "THIS IS MINE/OURS!"  The following statements refer to the feeling of owning the country The Netherlands together with other Dutch people. Please indicate to what extent you disagree or agree with the following statements. | When you think about your neighborhood and your neighbors, to what extent do you disagree or agree with the following statements? |
|  | 1 | I think this country is owned by us, the Dutch | I strongly feel like this is our neighborhood |
|  | 2 | I feel that this country belongs to us, the Dutch | It feels like this neighborhood is really ours |
|  | 3 | I have the feeling that we, the Dutch, own this country together |  |
|  | | | |
| Exclusive determination right | Intro | To what extent do you agree or disagree that Dutch people ... | To what extent do you disagree or agree with the following statements? |
|  | 1 | … have the exclusive right to determine matters that concern The Netherlands | My neighbors and I have the right to determine matters that concern our neighborhood |
|  | 2 | … have the exclusive right to determine who will be allowed in The Netherlands | It is up to me and my neighbors to determine what happens to our neighborhood |
|  | 3 | … have the exclusive right to determine what happens to The Netherlands in the future | My neighbors and I are in charge in our neighborhood |
|  | | | |
| Group responsibility | Intro | To what extent do you disagree or agree with the following statements? | To what extent do you disagree or agree with the following statements? |
|  | 1 | We the Dutch have a duty to take care of our country | It is my and my neighbors' duty to take care of our neighborhood |
|  | 2 | Together with other Dutch people I feel obliged to contribute to The Netherlands | Together with my neighbors I feel obliged to contribute to our neighborhood |
|  | 3 | We the Dutch are responsible for The Netherlands | My neighbors and I are responsible for our neighborhood |
|  | | | |
| Exclusion of outsiders | Intro | On a scale from 0% (definitely not) to 100% (definitely), what are the chances that you would do the following now or in the future? | Some people believe that their neighborhood should mainly be inhabited and used by original residents and that there should not be too many other people who come and live in their neighborhood. Others do not believe that.  On a scale from 0% (definitely not) to 100% (definitely), what are the chances that you would do the following in the future? |
|  | 1 | Vote for a political party that is committed to reducing immigration in The Netherlands | Convince other local residents that it is not good for the neighborhood when too many new people come and live here |
|  | 2 | Convince others that immigration is a problem | Support a local initiative that first offers vacant housing to current neighborhood residents |
|  | 3 | Participate in a protest against growing immigration in The Netherlands | Sign a petition to prevent too many new people from moving into your neighborhood |
|  | 4 |  | Help put up signs to prevent people who do not live in your neighborhood from making too much use of, for example, a playground or park in your neighborhood |
|  | | | |
| Stewardship behavior | Intro | Some people would like to contribute to The Netherlands, for example by doing voluntary work or by donating money. Other people need this less.  On a scale from 0% (definitely not) to 100% (definitely), what are the chances that in the future you will support a charity (by volunteering or donating money) that is committed to… | Some people like to actively contribute to their neighborhood, for example by doing voluntary work or donating money to neighborhood projects. Other people need this less.  On a scale from 0% (definitely not) to 100% (definitely), what are the chances that in the future you will actively commit to… |
|  | 1 | … maintain and preserve Dutch monuments and historic buildings | … reduce litter in your neighborhood |
|  | 2 | … support foundations, associations or healthcare institutions | ... maintain a flowerbed or garden in your neighborhood |
|  | 3 | … maintain and preserve Dutch natural landscapes | … preserve characteristic places or buildings in the neighborhood |
|  | 4 | … reduce litter in The Netherlands | ... keep the streets as clean as possible |
|  | | | |
| Group identification | Intro | For some people their Dutch identity is important, for others not. To what extent do you disagree or agree with the following statements? | The following questions are about your neighbors. To what extent do you disagree or agree with the following statements? |
|  | 1 | I strongly feel Dutch | I identify with other neighbors |
|  | 2 | Being Dutch is important to me | I often say "we" instead of "them" when I talk about residents of our neighborhood |
|  | 3 | I identify with other Dutch people | When someone criticizes residents of our neighborhood, I feel personally offended |
|  | | | |
| Place attachment | Intro | Some people feel strongly attached to the country where they live, others less so. To what extent do you disagree or agree with the following statements?  If you have never been abroad, try to imagine it. | To what extent do you disagree or agree with the following statements? |
|  | 1 | When I'm abroad for a while, I get homesick for The Netherlands | When I am gone, I miss my neighborhood |
|  | 2 | I would hate to move to another country | I would not want to move to another neighborhood |
|  | 3 | If I have been outside the country for a while, I am always happy to come back | When I have been away, I am always happy to come back to my neighborhood |

| **Appendix B.** Translations of the exact wording of all multi-item variables used in the four studies (continued) | | | |
| --- | --- | --- | --- |
|  |  | Study 3 | Study 4 |
| Collective psychological ownership | Intro | When you really imagine the situation, how much do you disagree or agree with the following statements? | When you really imagine the situation, how much do you disagree or agree with the following statements? |
|  | 1 | If I think about the park, I really feel that it is owned by us, neighbors | If I think about the park, I really feel that it is owned by us, neighbors |
|  | 2 | I have a strong feeling that this field belongs to us, neighbors | I have a strong feeling that this field belongs to us, neighbors |
|  | 3 |  |  |
|  | | | |
| Exclusive determination right | Intro | To what extent do you disagree or agree with the following statements? | To what extent do you disagree or agree with the following statements? |
|  | 1 | It is important to me that my neighbors and I get to decide whether or not to change the park | It is important that we neighbors get to decide whether or not to change the park |
|  | 2 | It is up to me and my neighbors to determine what happens in the park | It is up to us neighbors to determine what happens in the park |
|  | 3 | I find it important that my neighbors and I have the say in the park | I find it important that we neighbors have the say in the park |
|  | | | |
| Group responsibility | Intro | To what extent do you disagree or agree with the following statements? | To what extent do you disagree or agree with the following statements? |
|  | 1 | It is my and my neighbors' duty to take care of the park | It is the duty of us neighbors to take care of the park |
|  | 2 | Together with my neighbors I feel obliged to contribute to the park | We neighbors should feel obliged to contribute to the park |
|  | 3 | My neighbors and I are responsible for the park | We as a neighborhood are responsible for the park |
|  | | | |
| Exclusion of outsiders | Intro | Now imagine that the park is recently used more and more by people who do not live in your street.  On a scale from 0% (definitely not) to 100% (definitely), what are the chances that you would do the following? | Now imagine that the park is recently used more and more by people who do not live in your street.  On a scale from 0% (definitely not) to 100% (definitely), what are the chances that you as local residents together would do the following? |
|  | 1 | Place a sign that reads 'for local residents' | Place a sign that reads 'for local residents' |
|  | 2 | Place a small fence around the park | Place a small fence around the park |
|  | 3 | Together with your neighbors, try to monitor more | Together with your neighbors, try to monitor more |
|  | 4 | Make clear to others that they cannot just make use of the park | Make clear to others that they cannot just make use of the park |
|  | | | |
| Stewardship behavior | Intro | Some people would like to actively contribute to the park. Other people need this less.  On a scale from 0% (definitely not) to 100% (definitely), what are the chances that you … | In some neighborhoods people actively contribute to their neighborhood. In other neighborhoods this happens less.  On a scale from 0% (definitely not) to 100% (definitely), what are the chances that you as local residents together … |
|  | 1 | … will help to maintain the park | … will maintain the park |
|  | 2 | ... will clean up litter in the park | ... will clean up litter in the park |
|  | 3 | … will put in money for a new picnic table if the old one is broken | … will put in money for a new picnic table if the old one is broken |
|  |  |  |  |

**Appendix C.** Confidence intervals in all studies

In all three studies, we applied bootstrapping with 1,000 iterations and used a Maximum Likelihood (ML) estimator to calculate 95% confidence intervals. Using ML, as opposed to MLR in the main results, did not substantially change the results.

| **Table C-1.** Standardized 95% confidence intervals of the mediation model, Study 1 | | | | |
| --- | --- | --- | --- | --- |
|  | Exclusive determination right | Group responsibility | Exclusion of outsiders | Stewardship behavior |
| *Direct effects* |  |  |  |  |
| Collective psychological ownership | .456–.655 | .284–.520 | .027–.296 | -.118–.188 |
| Exclusive determination right |  |  | .104–.387 | -.186–.106 |
| Group responsibility |  |  | -.145–.116 | .068–.358 |
|  |  |  |  |  |
| Group identification | -.066–.153 | .207–.474 | -.139–.121 | -.125–.191 |
| Place attachment | -.075–.126 | -.104–.096 | -.058–.165 | -.155–.111 |
| Political orientation | -.086–.078 | -.073–.104 | .124–.367 | -.163–.085 |
| Sovereignty | .186–.375 | -.155–.025 | .066–.283 | -.259–-.023 |
| Philanthropy | -.126–.056 | .216–.426 | -.188–.079 | .018–.279 |
| Gender (female) | -.115–.013 | -.066–.086 | -.147–.022 | -.114–.064 |
| Age | -.159–-.017 | -.066–.086 | -.187–-.009 | -.020–.163 |
| Education level | -.055–.078 | -.039–.113 | -.061–.094 | -.006–.159 |
|  |  |  |  |  |
| *Indirect effects* |  |  |  |  |
| Collective psychological ownership > exclusive determination right |  |  | .055–.218 | -.104–.059 |
| Collective psychological ownership > group responsibility |  |  | -.060–.048 | .021–.150 |
|  |  |  |  |  |
| *Total effects* |  |  |  |  |
| Collective psychological ownership |  |  | .184–.399 | -.017–.213 |
|  |  |  |  |  |
| N | 617 |  |  |  |
|  | | | | |

| **Table C-2.** Standardized 95% confidence intervals of the mediation model, Study 2 | | | | |
| --- | --- | --- | --- | --- |
|  | Exclusive determination right | Group responsibility | Exclusion of outsiders | Stewardship behavior |
| *Direct effects* |  |  |  |  |
| Collective psychological ownership | .196–.449 | .102–.339 | -.360–-.087 | -.167–.052 |
| Exclusive determination right |  |  | .087–.319 | -.226–-.043 |
| Group responsibility |  |  | -.207–.070 | .391–.595 |
|  |  |  |  |  |
| Group identification | .186–.456 | .238–.490 | .297–.593 | .197–.455 |
| Place attachment | -.244–.028 | -.078–.214 | -.198–.089 | -.184–.081 |
| Gender (female) | -.069–.071 | -.065–.068 | -.165–-.028 | -.100–.028 |
| Age | -.092–.068 | -.043–.105 | -.149–.033 | -.122–.028 |
| Education level | -.062–.079 | .025–.156 | -.180–-.029 | .006–.137 |
| Mixed ethnic background | -.056–.085 | -.081–.077 | -.074–.067 | .005–.133 |
| Place of residence size |  |  |  |  |
| Large city | -.012–.150 | -.165–-.007 | -.058–.119 | -.049–.109 |
| Average city | -.039–.119 | -.080–.061 | -.041–.117 | -.074–.068 |
| Small city | -.060–.084 | -.064–.059 | -.042–.115 | -.075–.059 |
| Length of neighborhood residence | -.175–-.017 | -.156–-.021 | .013–.193 | -.093–.050 |
| Share of newcomers | -.046–.091 | -.081–.056 | -.017–.128 | -.072–.070 |
| Social cohesion | -.173–-.001 | -.032–.127 | -.084–.087 | -.020–.143 |
|  |  |  |  |  |
| *Indirect effects* |  |  |  |  |
| Collective psychological ownership > exclusive determination right |  |  | .017–.114 | -.077–-.009 |
| Collective psychological ownership > group responsibility |  |  | -.048–.018 | .042–.175 |
|  |  |  |  |  |
| *Total effects* |  |  |  |  |
| Collective psychological ownership |  |  | -.302–-.044 | -.104–.119 |
|  |  |  |  |  |
| N | 784 |  |  |  |
|  | | | | |

| **Table C-3.** Standardized 95% confidence intervals of the mediation model, Study 3 | | | | |
| --- | --- | --- | --- | --- |
|  | Exclusive determination right | Group responsibility | Exclusion of outsiders | Stewardship behavior |
| *Direct effects* |  |  |  |  |
| Ownership manipulation  (1 = ownership condition) | .029–.267 | .423–.594 | -.119–.150 | -.103–.110 |
| Exclusive determination right |  |  | .229–.545 | -.209–.051 |
| Group responsibility |  |  | .078–.369 | .629–.885 |
|  |  |  |  |  |
| *Indirect effects* |  |  |  |  |
| Ownership manipulation > exclusive determination right |  |  | .006–.109 | -.034–.010 |
| Ownership manipulation > group responsibility |  |  | .036–.191 | .287–.483 |
|  |  |  |  |  |
| *Total effects* |  |  |  |  |
| Ownership manipulation |  |  | .073–.300 | .276–.477 |
|  |  |  |  |  |
| N | 384 |  |  |  |
|  | | | | |

| **Table C-4.** Standardized 95% confidence intervals of the mediation model, Study 4 | | | | |
| --- | --- | --- | --- | --- |
|  | Exclusive determination right | Group responsibility | Exclusion of outsiders | Stewardship behavior |
| *Direct effects* |  |  |  |  |
| Ownership manipulation  (1 = ownership condition) | .151–.340 | .245–.419 | -.080–.101 | -.014–.149 |
| Exclusive determination right |  |  | .127–.449 | -.122–.081 |
| Group responsibility |  |  | .055–.343 | .559–.751 |
|  |  |  |  |  |
| *Indirect effects* |  |  |  |  |
| Ownership manipulation > exclusive determination right |  |  | .018–.130 | -.030–.020 |
| Ownership manipulation > group responsibility |  |  | .014–.127 | .150–.285 |
|  |  |  |  |  |
| *Total effects* |  |  |  |  |
| Ownership manipulation |  |  | .026–.264 | .187–.373 |
|  |  |  |  |  |
| N | 502 |  |  |  |
| **Appendix D.** Standardized regression coefficients for the mediation model, Study 1 | | | | |
|  | Exclusive determination right | Group responsibility | Exclusion of outsiders | Stewardship behavior |
| *Direct effects* |  |  |  |  |
| Collective psychological ownership | .556 (.051)*** | .402 (.057)*** | .161 (.066)* | .035 (.077) |
| Exclusive determination right |  |  | .246 (.068)*** | -.040 (.072) |
| Group responsibility |  |  | -.015 (.065) | .213 (.071)** |
|  |  |  |  |  |
| Group identification | .043 (.056) | .341 (.065)*** | -.009 (.067) | .033 (.080) |
| Place attachment | .026 (.050) | -.004 (.049) | .054 (.055) | -.022 (.060) |
| Political orientation | -.004 (.040) | .015 (.042) | .245 (.059)*** | -.039 (.058) |
| Sovereignty | .280 (.048)*** | -.065 (.048) | .174 (.052)** | -.141 (.056)* |
| Philanthropy | -.035 (.045) | .321 (.051)*** | -.055 (.065) | .148 (.063)* |
| Gender (female) | -.051 (.033) | -.011 (.036) | -.063 (.042) | -.025 (.044) |
| Age | -.088 (.036)* | .010 (.038) | -.098 (.046)* | .072 (.046) |
| Education level | .011 (.033) | .037 (.037) | .016 (.038) | .077 (.044) |
|  |  |  |  |  |
| *Indirect effects* |  |  |  |  |
| Collective psychological ownership > exclusive determination right |  |  | .136 (.039)** | -.022 (.040) |
| Collective psychological ownership > group responsibility |  |  | -.006 (.026) | .086 (.031)** |
|  |  |  |  |  |
| *Total effects* |  |  |  |  |
| Collective psychological ownership |  |  | .292 (.054)*** | .098 (.059) |
|  |  |  |  |  |
| R^2^ | .547 | .503 | .419 | .129 |
| N | 617 |  |  |  |
| * *p <* .05; ** *p <* .01; *** *p* < .001. | | | | |

| **Appendix E.** Standardized regression coefficients for a model without control variables, Study 1 | | | | |
| --- | --- | --- | --- | --- |
|  | Exclusive determination right | Group responsibility | Exclusion of outsiders | Stewardship behavior |
| *Direct effects* |  |  |  |  |
| Collective psychological ownership | .688 (.031)*** | .547 (.036)*** | .274 (.066)*** | -.034 (.070) |
| Exclusive determination right |  |  | .370 (.068)*** | -.135 (.065)* |
| Group responsibility |  |  | -.076 (.051) | .322 (.055)*** |
|  |  |  |  |  |
| *Indirect effects* |  |  |  |  |
| Collective psychological ownership > exclusive determination right |  |  | .254 (.048)*** | -.093 (.045)* |
| Collective psychological ownership > group responsibility |  |  | -.041 (.028) | .176 (.034)*** |
|  |  |  |  |  |
| *Total effects* |  |  |  |  |
| Collective psychological ownership |  |  | .487 (.038)*** | .050 (.047) |
|  |  |  |  |  |
| R^2^ | .473 | .300 | .309 | .079 |
| N | 617 |  |  |  |
| * *p <* .05; ** *p <* .01; *** *p* < .001. | | | | |

| **Appendix F.** Standardized regression coefficients for the mediation model of Study 2, including versions of the questionnaire as predictors of all endogenous variables | | | | |
| --- | --- | --- | --- | --- |
|  | Exclusive determination right | Group responsibility | Exclusion of outsiders | Stewardship behavior |
| *Direct effects* |  |  |  |  |
| Collective psychological ownership | .326 (.063)*** | .217 (.059)*** | -.222 (.065)** | -.059 (.055) |
| Exclusive determination right |  |  | .203 (.058)*** | -.134 (.045)** |
| Group responsibility |  |  | -.065 (.070) | .492 (.051)*** |
|  |  |  |  |  |
| Group identification | .323 (.068)*** | .367 (.063)*** | .438 (.073)*** | .326 (.064)*** |
| Place attachment | -.100 (.074) | .069 (.072) | -.050 (.071) | -.051 (.065) |
| Gender (female) | .000 (.036) | .000 (.033) | -.096 (.036)** | -.036 (.032) |
| Age | -.013 (.040) | .030 (.037) | -.058 (.046) | -.047 (.037) |
| Education level | .008 (.037) | .092 (.034)** | -.110 (.037)** | .072 (.033)* |
| Mixed ethnic background | .014 (.035) | -.003 (.040) | -.001 (.037) | .069 (.032)* |
| Place of residence size |  |  |  |  |
| Large city | .069 (.041) | -.085 (.039)* | .029 (.043) | .030 (.039) |
| Average city | .042 (.040) | -.008 (.036) | .037 (.038) | -.003 (.036) |
| Small city | .011 (.036) | -.004 (.032) | .039 (.038) | -.008 (.034) |
| Length of neighborhood residence | -.098 (.040)* | -.090 (.034)** | .104 (.045)* | -.023 (.037) |
| Share of newcomers | .024 (.034) | -.013 (.034) | .054 (.036) | -.001 (.036) |
| Social cohesion | -.087 (.042)* | .046 (.041) | .004 (.043) | .061 (.041) |
| Version (reference category = Version 1) |  |  |  |  |
| Version 2 | -.004 (.043) | .040 (.040) | -.079 (.042) | .009 (.040) |
| Version 3 | .055 (.042) | .039 (.041) | -.055 (.042) | .006 (.039) |
| Version 4 | -.009 (.042) | .031 (.037) | -.016 (.043) | .016 (.038) |
|  |  |  |  |  |
| *Indirect effects* |  |  |  |  |
| Collective psychological ownership > exclusive determination right |  |  | .066 (.024)** | -.044 (.017)** |
| Collective psychological ownership > group responsibility |  |  | -.014 (.016) | .107 (.032)** |
|  |  |  |  |  |
| *Total effects* |  |  |  |  |
| Collective psychological ownership |  |  | -.170 (.063)** | .004 (.057) |
|  |  |  |  |  |
| R^2^ | .227 | .385 | .197 | .393 |
| N | 784 |  |  |  |
| * *p <* .05; ** *p <* .01; *** *p* < .001. | | | | |

| **Appendix G.** Standardized regression coefficients for the mediation model, Study 2 | | | | |
| --- | --- | --- | --- | --- |
|  | Exclusive determination right | Group responsibility | Exclusion of outsiders | Stewardship behavior |
| *Direct effects* |  |  |  |  |
| Collective psychological ownership | .322 (.063)*** | .220 (.059)*** | -.223 (.065)** | -.058 (.055) |
| Exclusive determination right |  |  | .203 (.058)*** | -.134 (.045)** |
| Group responsibility |  |  | -.069 (.070) | .493 (.051)*** |
|  |  |  |  |  |
| Group identification | .321 (.068)*** | .364 (.063)*** | .445 (.072)*** | .326 (.063)*** |
| Place attachment | -.096 (.074) | .068 (.072) | -.054 (.071) | -.052 (.065) |
| Gender (female) | .001 (.035) | .001 (.033) | -.096 (.036)** | -.036 (.032) |
| Age | -.012 (.040) | .031 (.036) | -.058 (.046) | -.047 (.037) |
| Education level | .009 (.037) | .091 (.034)** | -.105 (.038)** | .072 (.033)* |
| Mixed ethnic background | .014 (.036) | -.002 (.040) | -.003 (.037) | .069 (.032)* |
| Place of residence size |  |  |  |  |
| Large city | .069 (.041) | -.086 (.039)* | .031 (.043) | .030 (.039) |
| Average city | .040 (.040) | -.009 (.036) | .038 (.038) | -.003 (.036) |
| Small city | .012 (.036) | -.003 (.031) | .036 (.038) | -.008 (.034) |
| Length of neighborhood residence | -.096 (.039)* | -.088 (.034)** | .103 (.046)* | -.022 (.037) |
| Share of newcomers | .023 (.033) | -.013 (.034) | .056 (.036) | -.001 (.036) |
| Social cohesion | -.087 (.042)* | .048 (.041) | .002 (.043) | .061 (.041) |
|  |  |  |  |  |
| *Indirect effects* |  |  |  |  |
| Collective psychological ownership > exclusive determination right |  |  | .066 (.024)** | -.043 (.016)* |
| Collective psychological ownership > group responsibility |  |  | -.015 (.016) | .108 (.032)** |
|  |  |  |  |  |
| *Total effects* |  |  |  |  |
| Collective psychological ownership |  |  | -.173 (.063)** | .007 (.057) |
|  |  |  |  |  |
| R^2^ | .224 | .384 | .192 | .393 |
| N | 784 |  |  |  |
| * *p <* .05; ** *p <* .01; *** *p* < .001. | | | | |

| **Appendix H. S**tandardized regression coefficients for a model without control variables, Study 2 | | | | |
| --- | --- | --- | --- | --- |
|  | Exclusive determination right | Group responsibility | Exclusion of outsiders | Stewardship behavior |
| *Direct effects* |  |  |  |  |
| Collective psychological ownership | .387 (.039)*** | .513 (.036)*** | -.045 (.054) | -.051 (.044) |
| Exclusive determination right |  |  | .228 (.056)*** | -.106 (.045)* |
| Group responsibility |  |  | .035 (.065) | .590 (.047)*** |
|  |  |  |  |  |
| *Indirect effects* |  |  |  |  |
| Collective psychological ownership > exclusive determination right |  |  | .088 (.024)*** | -.041 (.018)* |
| Collective psychological ownership > group responsibility |  |  | .018 (.033) | .302 (.035)*** |
|  |  |  |  |  |
| *Total effects* |  |  |  |  |
| Collective psychological ownership |  |  | .061 (.045) | .312 (.037)*** |
|  |  |  |  |  |
| R^2^ | .150 | .263 | .054 | .318 |
| N | 784 |  |  |  |
| * *p <* .05; ** *p <* .01; *** *p* < .001. | | | | |

**Appendix I.** Translations of the exact wording of the experiment, Study 3.

*Introduction to all respondents:*

We would like to ask you to read a short piece of text on the next page and to **really imagine the situation**. The situation may not correspond to your own neighborhood. But because we want to see how people react to neighborhood characteristics, it is important that you imagine the situation as good as possible. **Really take some time for this.**

*Ownership condition:*

| Imagine there is a little park in your street. It used to be just a piece of land where nothing happened, but you and your neighbors have tidied it up and put up a picnic table (see the photo). People living in your street use it a lot. You really have the feeling that it is 'your park’ and have given it the name 'our green park’ [‘ons groenplantsoen’].  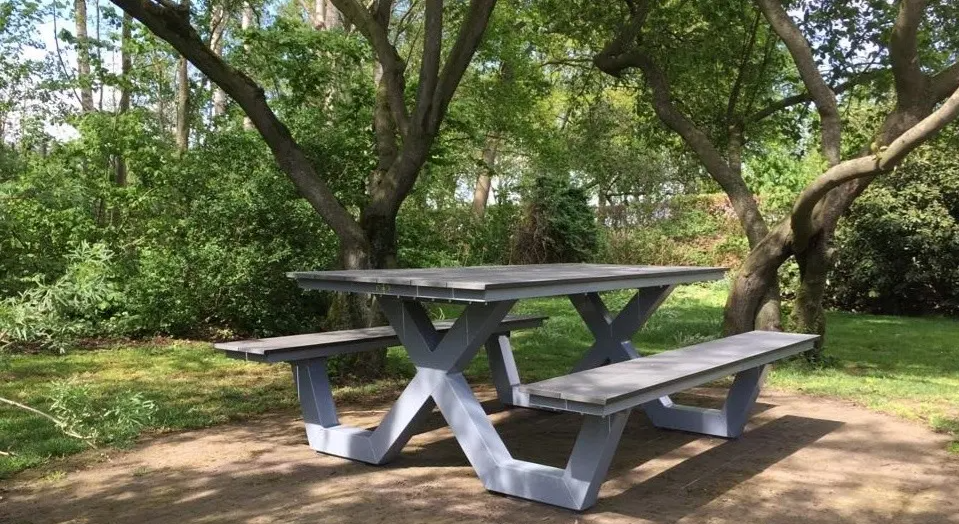 |
| --- |

*Control condition:*

| Imagine there is a little park in your street with a picnic table (see the photo). The people living in your street hardly use the park.  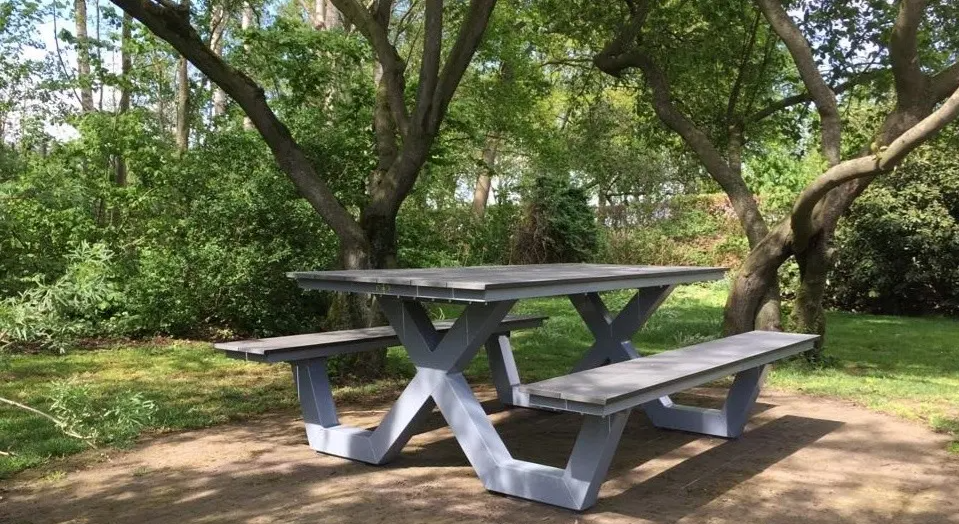 |
| --- |

*Note:* The photo used was taken by Houtcomposietdiscount.nl.

| **Appendix J.** Standardized regression coefficients for the mediation model, Study 3 | | | | |
| --- | --- | --- | --- | --- |
|  | Exclusive determination right | Group responsibility | Exclusion of outsiders | Stewardship behavior |
| *Direct effects* |  |  |  |  |
| Ownership manipulation  (1 = ownership condition) | .148 (.058)* | .509 (.045)*** | .016 (.069) | .003 (.053) |
| Exclusive determination right |  |  | .387 (.080)*** | -.079 (.062) |
| Group responsibility |  |  | .223 (.074)** | .757 (.063)*** |
|  |  |  |  |  |
| *Indirect effects* |  |  |  |  |
| Ownership manipulation > exclusive determination right |  |  | .057 (.025)* | -.012 (.010) |
| Ownership manipulation > group responsibility |  |  | .114 (.039)** | .385 (.049)*** |
|  |  |  |  |  |
| *Total effects* |  |  |  |  |
| Ownership manipulation |  |  | .186 (.059)** | .376 (.050)*** |
|  |  |  |  |  |
| R^2^ | .022 | .259 | .298 | .517 |
| N | 384 |  |  |  |
| * *p <* .05; ** *p <* .01; *** *p* < .001. | | | | |

**Appendix K.** Translations of the exact wording of the experiment, Study 4.

*Introduction to all respondents:*

We would like to ask you to read a short piece of text on the next page and to **really imagine the situation**. The situation may not correspond to your own neighborhood. But because we want to see how people react to neighborhood characteristics, it is important that you imagine the situation as good as possible. **Really take some time for this, otherwise it would unfortunately be a waste of effort.** You can only continue to the questions after 15 seconds.

*Ownership condition:*

| Imagine there is a little park in your street. It used to be just a piece of land where nothing happened, but the municipality has given it to the neighborhood and you and your neighbors have tidied it up and put up a picnic table (see the photo). You really have the feeling that it is 'your park’ and have given it the name 'our green park’ [‘ons groenplantsoen’].  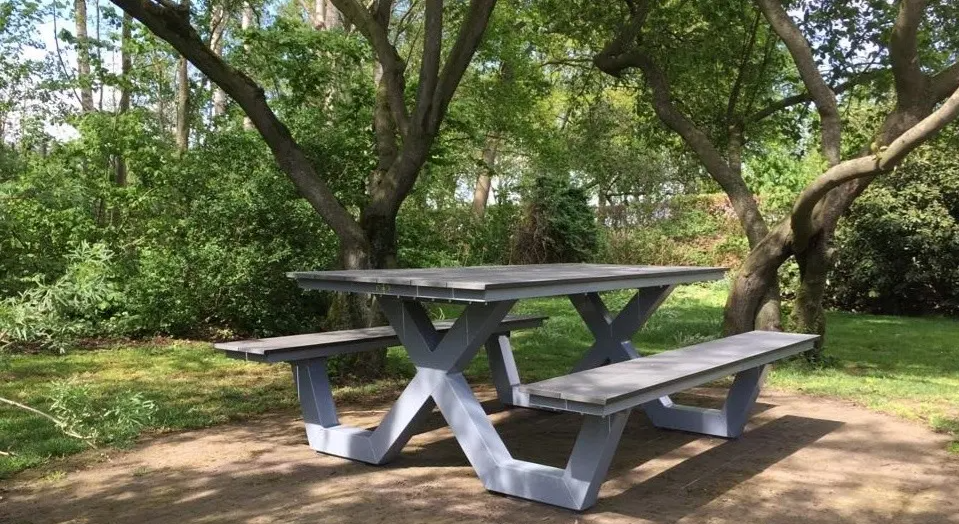 |
| --- |

*Control condition:*

| Imagine there is a little park in your street. It used to be just a piece of land where nothing happened, but the municipality has tidied it up and put up a picnic table (see the photo).  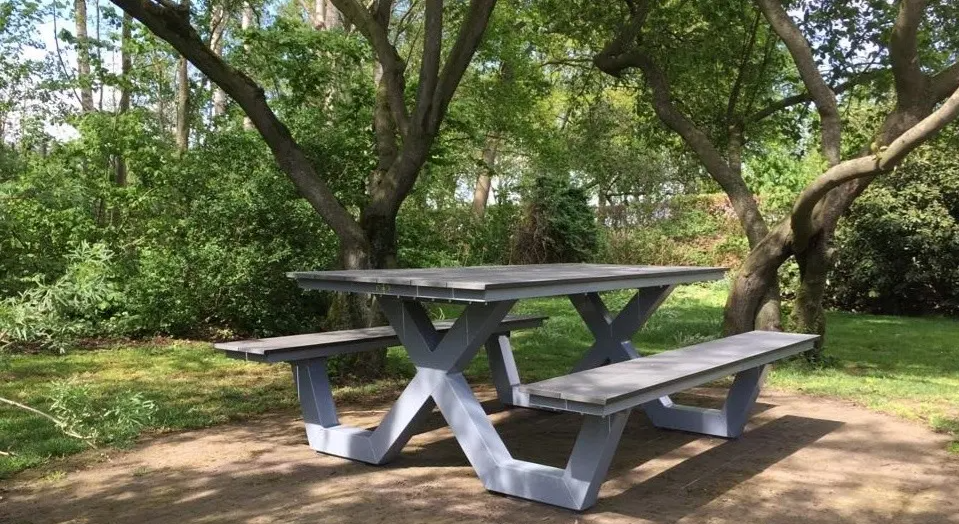 |
| --- |

*Note:* The photo used was taken by Houtcomposietdiscount.nl.

| **Appendix L.** Standardized regression coefficients for the mediation model, Study 4 | | | | |
| --- | --- | --- | --- | --- |
|  | Exclusive determination right | Group responsibility | Exclusion of outsiders | Stewardship behavior |
| *Direct effects* |  |  |  |  |
| Ownership manipulation  (1 = ownership condition) | .245 (.049)*** | .332 (.044)*** | .018 (.048) | .067 (.041) |
| Exclusive determination right |  |  | .302 (.089)** | -.021 (.052) |
| Group responsibility |  |  | .212 (.080)** | .655 (.049)*** |
|  |  |  |  |  |
| *Indirect effects* |  |  |  |  |
| Ownership manipulation > exclusive determination right |  |  | .074 (.028)** | -.005 (.013) |
| Ownership manipulation > group responsibility |  |  | .070 (.029)* | .218 (.034)*** |
|  |  |  |  |  |
| *Total effects* |  |  |  |  |
| Ownership manipulation |  |  | .163 (.049)** | .280 (.046)*** |
|  |  |  |  |  |
| R^2^ | .060 | .110 | .223 | .446 |
| N | 502 |  |  |  |
| * *p <* .05; ** *p <* .01; *** *p* < .001. | | | | |

1. As in Study 2, participants who were faster than one-third of the median duration of the full survey were considered speeders. [↑](#footnote-ref-1)
